# High endogenous activated protein C levels attenuates bleomycin‐induced pulmonary fibrosis

**DOI:** 10.1111/jcmm.12891

**Published:** 2016-06-14

**Authors:** Cong Lin, Jan von der Thüsen, Berend Isermann, Hartmut Weiler, Tom van der Poll, Keren Borensztajn, Chris A. Spek

**Affiliations:** ^1^ Center for Experimental and Molecular MedicineAcademic Medical CenterAmsterdamThe Netherlands; ^2^ Department of PathologyErasmus Medical CenterRotterdamThe Netherlands; ^3^ Medizinische FakultätOtto‐von‐Guericke‐Universität MagdeburgMagdeburgGermany; ^4^ Department of PhysiologyBloodCenter of WisconsinMilwaukeeWIUSA; ^5^ Inserm UMR1152Medical School Xavier BichatParisFrance; ^6^ Département Hospitalo‐universtaire FIRE (Fibrosis Inflammation and Remodeling) and LabEx InflamexParisFrance

**Keywords:** activated protein C, idiopathic pulmonary fibrosis, bleomycin and macrophages

## Abstract

Coagulation activation accompanied by reduced anticoagulant activity is a key characteristic of patients with idiopathic pulmonary fibrosis (IPF). Although the importance of coagulation activation in IPF is well studied, the potential relevance of endogenous anticoagulant activity in IPF progression remains elusive. We assess the importance of the endogenous anticoagulant protein C pathway on disease progression during bleomycin‐induced pulmonary fibrosis. Wild‐type mice and mice with high endogenous activated protein C APC levels (APC ^high^) were subjected to bleomycin‐induced pulmonary fibrosis. Fibrosis was assesses by hydroxyproline and histochemical analysis. Macrophage recruitment was assessed immunohistochemically. *In vitro*, macrophage migration was analysed by transwell migration assays. Fourteen days after bleomycin instillation, APC ^high^ mice developed pulmonary fibrosis to a similar degree as wild‐type mice. Interestingly, Aschcroft scores as well as lung hydroxyproline levels were significantly lower in APC ^high^ mice than in wild‐type mice on day 28. The reduction in fibrosis in APC ^high^ mice was accompanied by reduced macrophage numbers in their lungs and subsequent *in vitro* experiments showed that APC inhibits thrombin‐dependent macrophage migration. Our data suggest that high endogenous APC levels inhibit the progression of bleomycin‐induced pulmonary fibrosis and that APC modifies pulmonary fibrosis by limiting thrombin‐dependent macrophage recruitment.

## Introduction

Idiopathic pulmonary fibrosis (IPF) is a specific form of fibrosing idiopathic interstitial pneumonia, characterized by progressive and irreversible pathological changes, with a median survival of 3 years 1, 2. IPF comprises a group of conditions characterized by the formation and proliferation of (myo)fibroblast foci and exaggerated extracellular matrix (ECM) accumulation 3. The current pathogenesis paradigm suggests that pulmonary fibrogenesis results from an uncontrolled wound healing response that is initiated after repeated epithelium injury 4.

Beyond its primary role in hemostasis, coagulation activation in response to tissue injury seems to be a critical contributor in the pathogenesis of fibrotic lung disorders 5. A hypercoagulable state is commonly observed in IPF patients 6, 7, and coagulation factors, such as tissue factor (TF), factor (F)VII, FXa and thrombin, are increased in these patients. All these individual coagulation factors exert profibrotic cellular effects through activation of the cell surface protease‐activated receptors (PARs). Indeed, FVIIa may contribute to the development and/or progression of IPF by activating PAR‐2, whereas FXa induces profibrotic effects *via* either PAR‐1 or PAR‐2 8, 9, 10, 11. Thrombin, as the best‐described profibrotic coagulation factor, activates PAR‐1 leading to myofibroblast accumulation and subsequent fibrotic responses of lung (myo)fibroblasts, such as proliferation, migration and ECM synthesis (e.g. collagen) 12, 13, 14. The potential importance of coagulation factors in IPF is underscored by the fact that inhibiting coagulation limits pulmonary fibrosis in pre‐clinical experimental animal models 10, 15, 16, 17.

The hypercoagulable state observed in IPF patients may not only be due to increased coagulation factor expression but may also at least in part results from reduced anticoagulant activity. Indeed, the balance between pro‐ and anticoagulant pathways is compromised in patients with IPF, and especially the anticoagulant protein C pathway seems down regulated 18. Protein C, once activated by the thrombin‐thrombomodulin complex, prevents excessive coagulation *via* inactivation of factors Va and VIIIa 19, 20. Next to inhibiting coagulation, activated protein C (APC) exhibits anti‐inflammatory and vascular protective effects through PAR‐1, the same receptor activated by thrombin 21. In the context of lung injury, endogenous APC inhibits infection‐induced coagulation activation 22 and APC overexpression modifies neutrophil recruitment during experimental pneumococcal pneumonia 23. Particularly relevant for this study, exogenous APC instillation seems to limit bleomycin‐induced pulmonary fibrosis probably through its anti‐inflammatory activity 24. Surprisingly, however, low endogenous APC levels do not aggravate bleomycin‐induced pulmonary fibrosis although the lack of endogenous APC does provoke bleomycin‐induced bleeding into the pleural cavity with evident signs of pulmonary haemorrhage 25. Whether these apparent conflicting results of high *versus* low APC levels is due to exogenous *versus* endogenous manipulation of APC levels remains elusive. In this study, we consequently aimed to assess the significance of enhanced activity of the endogenous anticoagulant protein C pathway in IPF. To this end, we subjected mice with different endogenous APC levels to the pre‐clinical bleomycin induced pulmonary fibrosis model.

## Materials and methods

### Animal model of pulmonary fibrosis

Wild‐type C57Bl/6 mice were purchased from Charles River (Someren, the Netherlands). APC^high^ mice, with plasma APC levels almost forty times higher than in wild‐type mice, were generated and backcrossed to a C57BL/6 genetic background as described 26 and bred at the animal care facility of the Academic Medical Center. Endogenous overexpression of APC in APC^high^ mice has been confirmed in previous reports 26 as well as in our own laboratory 27. All procedures were performed on 8–10‐week‐old mice, and in accordance with the Institutional Standards for Humane Care and Use of Laboratory Animals. Experiments were approved by the Animal Care and Use Committee of the Academic Medical Center (Amsterdam, the Netherlands). Bleomycin (Sigma‐Aldrich, St. Louis, MO, USA) was administered by intranasal instillation (1 mg/kg bw) under anaesthesia. Mice were killed 14 or 28 days after bleomycin instillation, following which the left lungs were excised for histological analysis whereas the right lungs were homogenized for hydroxyproline and cytokine assays.

### Cells and reagents

Murine NIH3T3 fibroblasts and RAW264.7 macrophages were cultured in DMEM and IMDM, respectively, supplemented with 10% foetal calf serum (FCS). Cells were grown at 37°C in an atmosphere of 5% CO_2_. Unless indicated otherwise, cells were washed twice with PBS and serum starved for 4 hrs before stimulation. Thrombin was from Sigma‐Aldrich (St. Louis, MO, USA), recombinant human APC (rhAPC; Xigris) was obtained from Eli Lilly (Houten, the Netherlands) and recombinant mouse monocyte chemotactic protein (MCP‐1) was from (R&D systems, Minneapolis, MN, USA).

### Cell viability assays

Cells were seeded in 96‐well plates at a concentration of 5000 cells/well after which cell viability was determined using a 3‐(4,5‐dimethylthiazol‐2‐yl)‐2,5‐diphenyltetrazolium (MTT) assay at 24 hrs according to routine procedures 28.

### Wound scratch assay

Scratch assays were performed essentially as described before 29. In detail, fibroblasts were seeded in six‐well plates in DMEM supplemented with 10% FCS. After the cells formed a confluent monolayer, a scratch was created in the centre of the monolayer with a sterile p200 pipette tip. Next, medium was removed and cells were washed with serum‐free medium to remove floating debris. The cells were subsequently incubated for 18 hrs with serum‐free medium without (negative control) or with 10 nM thrombin or APC (concentrations of thrombin and APC are based on literature 30, 31, 32, 33. The ability of cells to close the wound was assessed by comparing the 0‐ and 18‐hr phase‐contrast micrographs of six marked points along the wounded area. The percentage of non‐recovered wound area was calculated by dividing the non‐recovered area after 18 hrs by the initial area at 0 hr.

### Western blot

Fibroblasts were seeded in 12‐well plates in DMEM supplemented with 10% FCS. After serum starvation for 4 hrs, the cells were incubated with serum‐free medium (negative control) with or without 10 nM thrombin or APC (concentrations of thrombin and APC are based on literature 26, 30, 31, 34. Twenty‐four hours later, cells were lysed in Laemmli lysis buffer and Western blots were performed as described before 29. In brief, protein samples were separated by 10% SDS gel electrophoresis and transferred to a PVDF membrane (Millipore, Billerica, MA, USA). Membranes were blocked for 1 hr in 4% milk in TBST and incubated overnight with monoclonal antibodies against α‐smooth muscle actin (α‐SMA), GAPDH (both Santa Cruz, CA, USA) or collagen (SouthernBiotech, Birmingham, AL, USA) at 4°C. All secondary antibodies were horseradish peroxidase (HRP)‐conjugated from DakoCytomation (Glostrup, Denmark) and diluted according to the manufacturer's instructions. Blots were imaged using Lumilight plus ECL substrate from Roche (Almere, the Netherlands) on an ImageQuant LAS 4000 biomolecular imager from GE Healthcare (Buckinghamshire, U.K).

### Transwell migration assays

Serum starved 2 × 10^4^ RAW264.7 cells were transferred to 8 μm pore‐size Cell Culture inserts coated with 0.1% (w/v) collagen. The cells were incubated in serum‐free medium with or without thrombin/APC (both 10 nM), and the inserts were incubated at 37°C for 10 hrs in serum‐free medium with MCP‐1 (50 ng/ml) as chemoattractant. For microscopic analysis, cells on the upper side of the Transwell membrane were removed with a cotton swab after which the inserts were fixed and stained in a crystal violet solution as described 32. The membranes were subsequently mounted on a glass slide, and migrated cells were counted by light microscopy. Cells were counted in five different fields using a 200× magnification.

### (Immuno)histological analysis

Four‐μm sections were deparaffinized and rehydrated. Slides were stained with haematoxylin and eosin (haematoxylin and eosin) according to routine procedures. In haematoxylin and eosin stainings, the severity of fibrosis was assessed according to the Ashcroft scoring system using a 100× magnification as described before 29. Two independent observers, blinded to the treatment group, scored the average Ashcroft score of 10 randomly selected fields of each lung section as calculated by averaging the individual field scores. For F4/80 staining, endogenous peroxidase activity was quenched with 0.3% H_2_O_2_ in methanol and the F4/80 antibody was incubated for 24 hrs at 4°C (1:500, AbD Serotec, Kidlington, UK). A horseradish peroxidase‐conjugated polymer detection system (ImmunoLogic, Duiven, the Netherlands) was applied for visualization, using an appropriate secondary antibody and diaminobenzidine (DAB) staining. Slides were photographed with a microscope equipped with a digital camera (Leica CTR500, Leica Microsystems, Wetzlar, Germany). Pictures of F4/80 staining were taken to cover the entirety of all sections. Colour intensity of stained areas was analysed semi‐quantitatively with ImageJ and expressed as percentage of the surface area essentially as described 35.

### ELISA

Active transforming growth factor‐beta 1 (TGF‐β1) was measured using a Mouse DuoSet kit (R&D systems, UK) as per the manufacturer's instructions.

### Hydroxyproline assay

Hydroxyproline analysis was performed by the hydroxyproline assay kit as per the manufacturer's instructions (Sigma‐Aldrich, the Netherlands) and as described before 29.

### Statistics

Statistical analyses were conducted using GraphPad Prism version 5.00 (GraphPad software, San Diego, CA, USA). Data were expressed as means ± SEM. Comparisons between two conditions were analysed using two‐tailed unpaired *t*‐tests when the data were normally distributed, otherwise Mann–Whitney analysis was performed. *P* < 0.05 were considered significant.

## Results

### Fibrosis progression is reduced in APC^high^ mice during bleomycin‐induced pulmonary fibrosis

**Figure 1:**
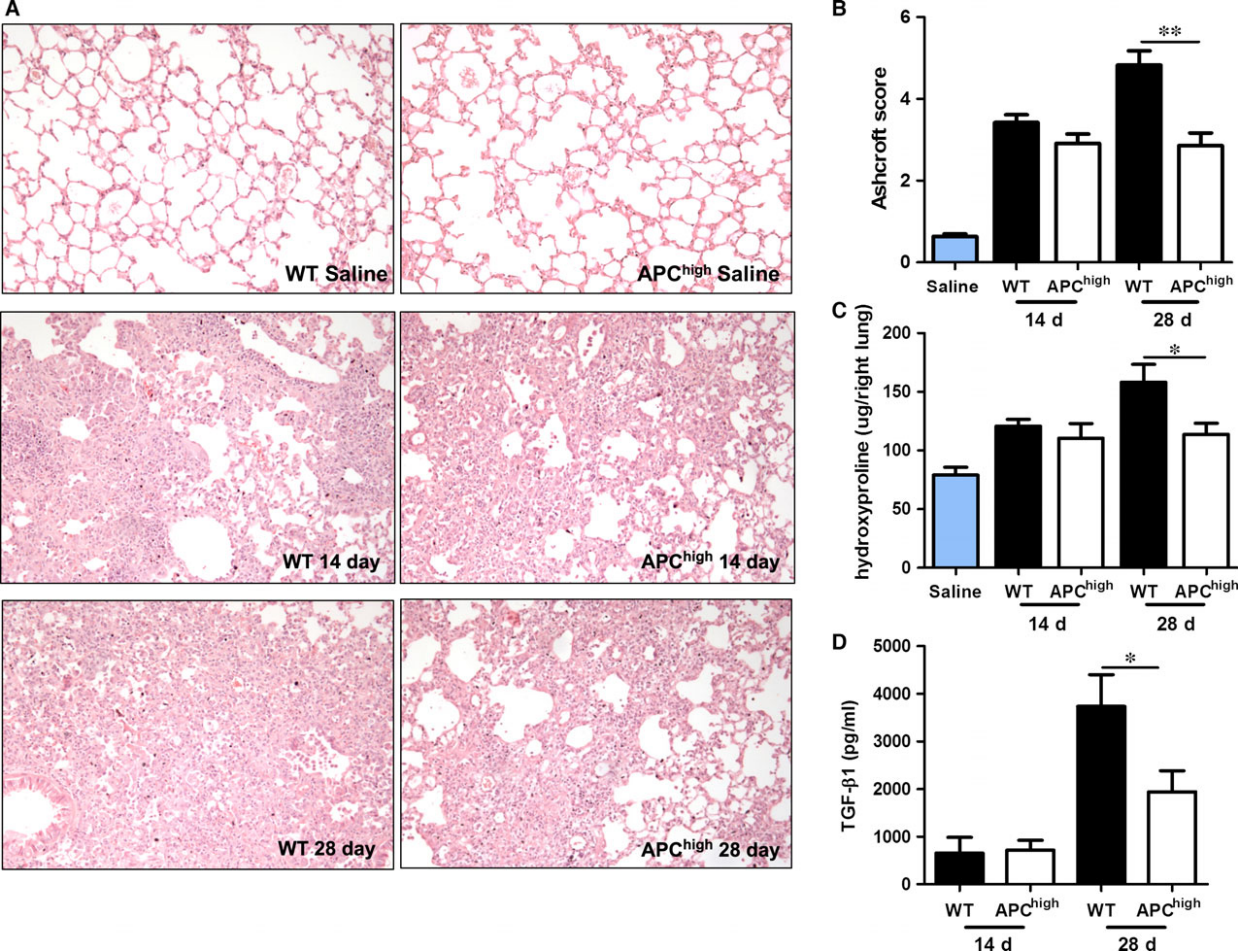
High endogenous APC levels limit the progression of pulmonary fibrosis. (**A**) Representative pictures of lungs of mice 14 and 28 days after saline or bleomycin instillation (100× magnification). (**B**) Quantification of pulmonary fibrosis 14 and 28 days after bleomycin instillation using the Ashcroft score. (**C**) Collagen content in lung homogenates obtained 14 or 28 days after bleomycin instillation. (**D**) Transforming growth factor (TGF)‐β1 levels in lung homogenates obtained 14 or 28 days after bleomycin instillation. Data are expressed as mean ± SEM (*n* = 8 per group, *P < 0.05 and **P < 0.01).

To study the effect of high endogenous APC levels during the progression of pulmonary fibrosis, APC^high^ and wild‐type mice were subjected to bleomycin‐induced fibrosis for either 14 or 28 days. APC^high^ mice express a hyperactivatable form of APC (D167F/D172K) 26, which results in high endogenous plasma APC levels (18 ng/ml in our mouse colony *versus* undetectable levels in wild‐type controls 27). As shown in Figure 1, bleomycin‐induced extensive patchy areas of fibrosis were present to a similar extent in both wild‐type and APC^high^ mice on day 14. During disease progression, the inflammatory and fibrotic effects culminated in severe pulmonary fibrosis at day 28 in wild‐type mice (Fig. 1A). Interestingly, however, the increase in pulmonary fibrosis over time was not observed in APC^high^ mice, and indeed Ashcroft scores were similar in APC^high^ mice at day 14 and day 28 (Fig. 1B). In line with the Ashcroft scores, lung hydroxyproline levels did not show differences between APC^high^ and wild‐type mice on day 14, whereas these levels were significantly higher in wild‐type mice at 28 days after bleomycin instillation (Fig. 1C). Similar to Ashcroft scores and hydroxyproline levels, TGF‐β1 concentrations are relatively low in wild‐type and APC^high^ mice at day 14. During disease progression, TGF‐β1 levels increase in both wild‐type and APC^high^ mice although the increase is clearly reduced in APC^high^ mice at day 28 (Fig. 1D). Overall, these results show that high endogenous APC levels limit disease progression during bleomycin‐induced pulmonary fibrosis.

### APC inhibits thrombin‐induced monocyte/macrophage recruitment during pulmonary fibrosis

**Figure 2:**
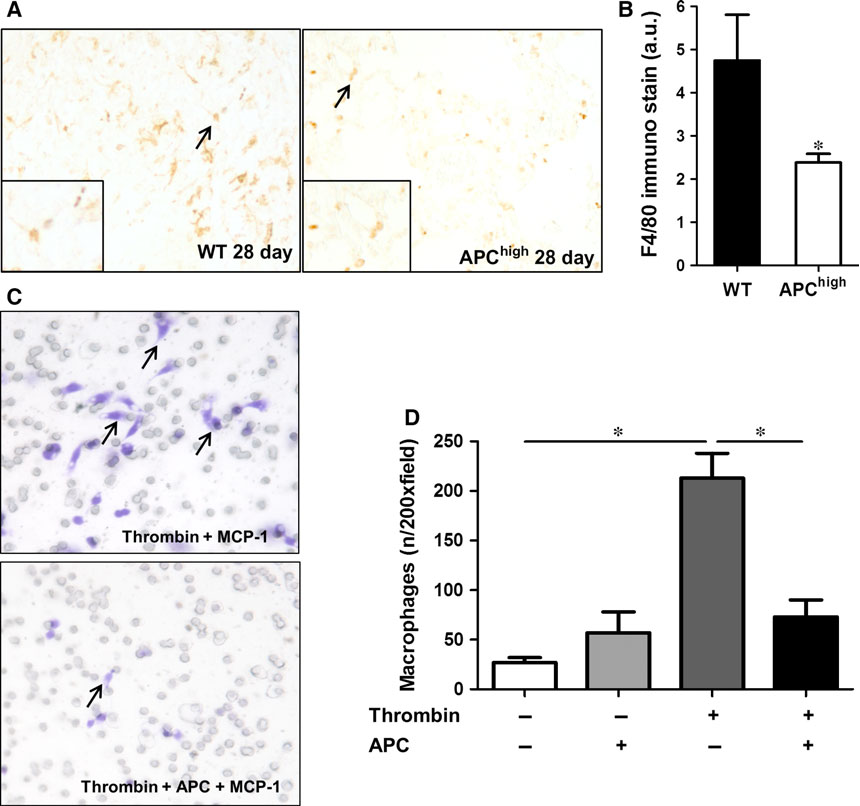
Effect of high endogenous APC levels on macrophage recruitment. (**A**) Representative pictures of F4/80 stained lung sections obtained 28 days after bleomycin instillation in wild‐type (WT) and APC ^high^ mice (100× magnification). The arrows point to (examples of) F4/80 positive macrophages. (**B**) Quantification of macrophage numbers in mice 28 days after bleomycin instillation. (mean ± SEM,* n* = 8 per group, *P < 0.05). (**C**) Representative pictures of RAW264.7 cell migration towards MCP‐1 after stimulation with thrombin (10 nM) in the presence or absence of rhAPC (10 nM). The arrows indicate examples of stained macrophages. (**D**) Quantification of the data presented in C (mean ± SEM of an experiment performed three times, *P < 0.05). APC, activated protein C.

Macrophage recruitment in response to inflammatory mediators produced by injured epithelial cells is a key process in fibrosis 36. Moreover, we recently showed that thrombin‐dependent PAR‐1 signalling potentiates macrophage migration towards bleomycin‐treated epithelial cells, thereby driving pulmonary fibrosis 34. Consequently, we determined macrophage numbers in fibrotic lungs of both wild‐type and APC^high^ mice. As shown in Figure 2A, F4/80 positive macrophages were diffusely present in lungs of wild‐type mice 28 days after bleomycin instillation. In APC^high^ mice, macrophage numbers were reduced by around 50% as compared to wild‐type mice (Fig. 2A–B). Interestingly, the reduced macrophage numbers in APC^high^ mice at day 28 after bleomycin instillation were not due to a direct effect of APC on macrophage migration: as shown in Figure 2C, RAW264.7 macrophage migration towards MCP‐1 was not modified by APC treatment *in vitro*. APC did however (almost) completely prevent thrombin‐induced RAW264.7 cell migration towards MCP‐1 (Fig. 2D). Together, the results suggest that APC inhibits thrombin‐induced macrophage migration during pulmonary fibrosis.

### APC does not interfere with thrombin‐induced profibrotic effects on fibroblasts

**Figure 3:**
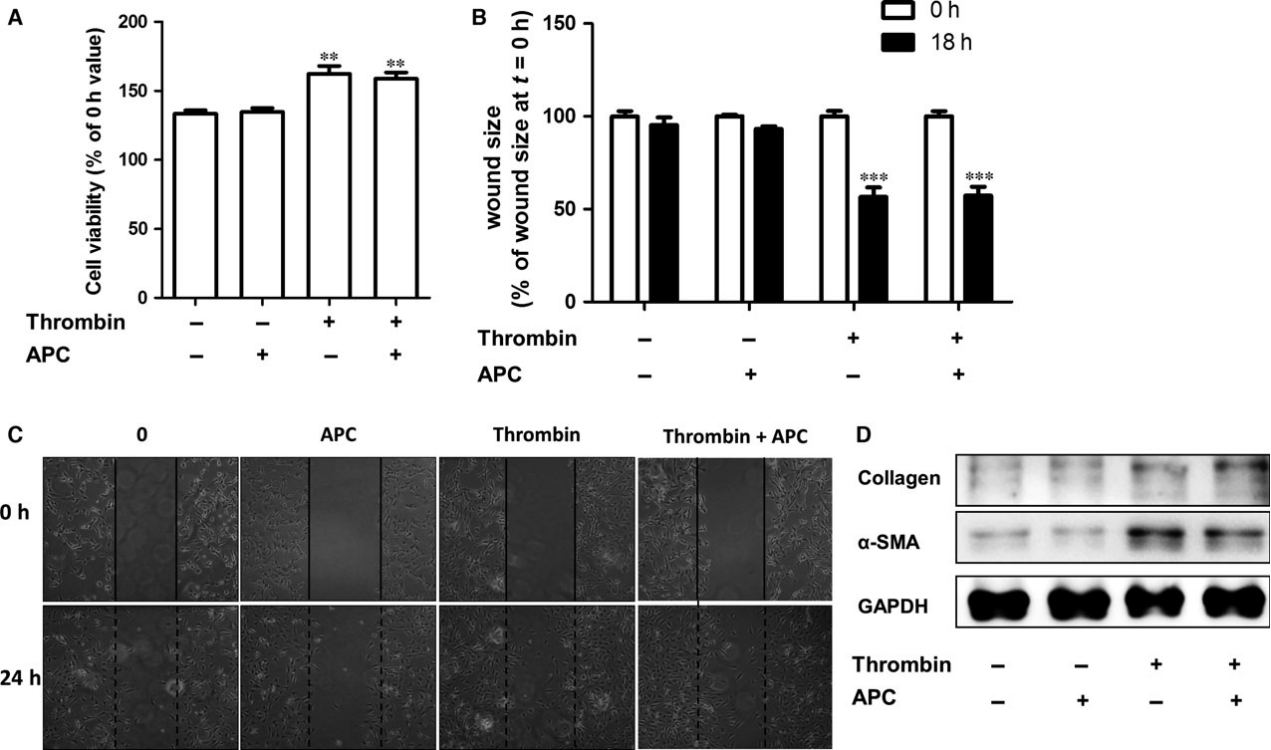
APC does not interfere with thrombin‐induced profibrotic effects on fibroblasts. (**A**) Cell viability of NIH3T3 cells treated with thrombin and/or APC (both 10 nM) as evaluated by MTT assays (mean ± SEM of an experiment performed two times in octoplo, **P < 0.01). (**B**) Quantification of the results depicted in (**C**). Data are expressed as mean ± SEM (*n* = 6). ***P < 0.001. (**C**) Wound size of NIH3T3 fibroblast monolayers after treatment with PBS (control), rhAPC (10 nM), thrombin (10 nM) or the combination of thrombin and rhAPC for 18 hrs. Shown are photographs of representative microscopic fields. (**D**) Western blot analysis of α‐smooth muscle actin and collagen expression in NIH3T3 cells stimulated with thrombin and/or APC (both 10 nM). GAPDH served as loading control. APC, activated protein C.

Thrombin induces several profibrotic processes on fibroblasts, such as fibroblast proliferation, migration, differentiation and ECM production 13, 16. Therefore, we assessed whether APC may, next to reducing thrombin‐dependent macrophage recruitment, also modify pulmonary fibrosis by inhibiting thrombin‐dependent profibrotic responses in fibroblasts. As shown in Figure 3A–B, as opposed to APC treatment, thrombin stimulation increased fibroblast proliferation and migration. Interestingly, APC did not modify these thrombin‐induced profibrotic responses. In line, APC also did not modify thrombin‐induced fibroblast differentiation and ECM synthesis (Fig. 3C). Hence, APC neither directly affects fibrotic responses of fibroblasts nor does it limit thrombin‐induced profibrotic effects of fibroblasts.

## Discussion

Coagulation activation is a frequent phenomenon in IPF and IPF patients are more than four times more likely to have a hypercoagulable state than general population controls 5, 7, 18. The presence of a hypercoagulable state is not only associated with disease severity at diagnosis but also adversely impacts on survival of IPF patients 7. At least in part, hypercoagulability in IPF patients may be due to reduced anticoagulant activity, and here we consequently addressed the importance of the endogenous protein C pathway. We show that endogenous APC modifies disease progression and affords protection against bleomycin‐induced pulmonary fibrosis.

Mice expressing high endogenous APC levels (i.e. APC^high^ mice) are protected from bleomycin‐induced pulmonary fibrosis as evident from reduced Ashcroft scores, hydroxyproline concentrations and TGF‐β1 levels at day 28 after bleomycin instillation. The reduction in fibrosis in APC^high^ mice was accompanied by significantly decreased macrophage numbers in their lungs. This may be particularly important as macrophage recruitment in response to lung epithelial cell injury is a key process in pulmonary fibrosis. Recruited macrophages produce profibrotic cytokines like TGF‐β that activate fibroblasts, thereby potentiating their profibrotic responses 4, 36.

It is tempting to speculate that the reduction in pulmonary fibrosis observed at day 28 is explained by a direct inhibitory effect of endogenous APC on macrophage recruitment. Indeed, APC has previously been shown to inhibit migration of lymphocytes towards IL‐8, RANTES and MCP‐1 33 and to limit migration and activation of rheumatoid arthritis monocytes *via* the endothelial protein C receptor (EPCR) 37. Here, we show that APC does not directly inhibit migration of RAW264.7 macrophages towards MCP‐1 by itself but instead blocks thrombin‐induced macrophage migration. Most likely, APC competes for PAR‐1 cleavage, thereby limiting thrombin‐dependent PAR‐1 signalling and subsequent macrophage migration. Such competition between APC and thrombin is well‐known and APC‐ or thrombin‐induced PAR‐1 cleavage leads to distinct or even opposite downstream signalling events. For example, APC switches thrombin‐induced PAR‐1 signalling from a disruptive to a protective effect in human umbilical vein endothelial cells 21, 38.

As opposed to modifying thrombin‐induced macrophage migration, APC did not affect thrombin‐induced profibrotic responses of fibroblasts, like fibroblast proliferation, migration, differentiation and collagen deposition. This may be surprising at a first glance, but this is most likely explained by the fact that fibroblasts do not express EPCR, which is essential for APC‐dependent PAR‐1 cleavage 39, 40.

In line with our data showing that endogenous APC limits bleomycin‐induced pulmonary fibrosis, intratracheal administration of exogenous human APC reduced the progression of pulmonary fibrosis as well 24. Interestingly however, exogenous APC seemed more effective in reducing fibrosis at an earlier time‐point as hydroxyproline levels were already reduced 14 days after bleomycin infusion in case of exogenous APC administration. Although we do not have a definitive explanation for the increased efficacy of exogenous APC, it may well be due to higher initial concentrations of exogenous *versus* endogenous APC. Irrespective of the precise underlying molecular mechanisms, both studies emphasize the importance of the anticoagulant protein C pathway in disease progression of IPF and the availability of endogenous APC may thus be an important clinical and pharmacological parameter in patients with IPF. Consequently, preservation and/or restoration of endogenous APC generation might be an interesting target for limiting IPF progression.

In conclusion, this study reveals that endogenous APC inhibits the progression of bleomycin‐induced pulmonary fibrosis. We suggest that APC limits pulmonary fibrosis due to the inhibitory effect of APC on thrombin‐induced macrophage recruitment rather than any direct antifibrotic effect of APC on fibroblasts.

## Conflict of interest

The authors confirm that there are no conflicts of interest.
